# A pilot study: sequential gemcitabine/cisplatin and icotinib as induction therapy for stage IIB to IIIA non-small-cell lung adenocarcinoma

**DOI:** 10.1186/1477-7819-11-96

**Published:** 2013-04-26

**Authors:** Chao Lv, Yuanyuan Ma, Qin Feng, Fang Fang, Hua Bai, Bingtian Zhao, Shi Yan, Nan Wu, Qingfeng Zheng, Shaolei Li, Jinfeng Chen, Jia Wang, Yuan Feng, Yuzhao Wang, Yuquan Pei, Jian Fang, Yue Yang

**Affiliations:** 1 Department of Thoracic Surgery II, Key Laboratory of Carcinogenesis and Translational Research(Ministry of Education), Peking University Cancer Hospital and Institute, 52 Fucheng Road, Beijing, Haidian District, 100142, China; 2 Department of Pathology, Key Laboratory of Carcinogenesis and Translational Research(Ministry of Education), Peking University Cancer Hospital and Institute, 52 Fucheng Road, Beijing, Haidian District, 100142, China; 3 Department of Thoracic Medical Oncology I, Key Laboratory of Carcinogenesis and Translational Research (Ministry of Education), Peking University Cancer Hospital and Institute, 52 Fucheng Road, Beijing, Haidian District, 100142, China; 4 Department of Thoracic Medical Oncology II, Key Laboratory of Carcinogenesis and Translational Research (Ministry of Education), Peking University Cancer Hospital and Institute, 52 Fucheng Road, Beijing, Haidian District, 100142, China

**Keywords:** Non-small-cell lung cancer, Sequential, Induction chemotherapy, EGFR mutation, Adenocarcinoma

## Abstract

**Background:**

A phase II clinical trial previously evaluated the sequential administration of erlotinib after chemotherapy for advanced non-small-cell lung cancer (NSCLC). This current pilot study assessed the feasibility of sequential induction therapy in patients with stage IIB to IIIA NSCLC adenocarcinoma.

**Methods:**

Patients received gemcitabine 1,250 mg/m^2^ on days 1 and 8 and cisplatin 75 mg/m^2^ on day 1, followed by oral icotinib (125 mg, three times a day) on days 15 to 28. A repeatcomputed tomography(CT) scan evaluated the response to the induction treatment after two 4-week cycles and eligible patients underwent surgical resection. The primary objective was to assess the objective response rate (ORR), while EGFR and KRAS mutations and mRNA and protein expression levels of ERCC1 and RRM1 were analyzed in tumor tissues and blood samples.

**Results:**

Eleven patients, most with stage IIIA disease, completed preoperative treatment. Five patients achieved partial response according to the Response Evaluation Criteria in Solid Tumors (RECIST) criteria (ORR=45%) and six patients underwent resection. Common toxicities included neutropenia, alanine transaminase (ALT) elevation, fatigue, dry skin, rash, nausea, alopecia and anorexia. No serious complications were recorded perioperatively. Three patients had exon 19 deletions and those with EGFR mutations were more likely to achieve a clinical response (*P*= 0.083). Furthermore, most cases who achieved a clinical response had low levels of ERCC1 expression and high levels of RRM1.

**Conclusions:**

Two cycles of sequentially administered gemcitabine/cisplatin with icotinib as an induction treatment is a feasible and efficacious approach for stage IIB to IIIA NSCLC adenocarcinoma, which provides evidence for the further investigation of these chemotherapeutic and molecularly targeted therapies.

## Background

Stage IIB to IIIA non-small-cell lung cancer (NSCLC) has an unfavorable prognosis, with a low 5-year survival rate that ranges from 19% to 25% using clinical stage prediction, and a high recurrence rate despite aggressive surgical management [1,2]. Numerous trials have investigated the use of perioperative chemotherapy as a strategy to improve survival. A pooled analysis by the Lung Adjuvant Cisplatin Evaluation (LACE) Collaborative Group presented a 5-year absolute benefit of 5.4% using postoperative cisplatin-based chemotherapy, especially for stage II to III cases of NSCLC (hazard ratio (HR) = 0.83) [3]; while for the induction therapy, the Chemotherapy in Early Stages Trial (ChEST) yielded a significant improvement in terms of both disease-free survival (DFS; HR = 0.51) and overall survival (OS; HR = 0.42) of patients with clinical stage IIB to IIIA NSCLC using preoperative gemcitabine plus cisplatin [4].

Over the last decade, the development of targeted therapies have held great promise for the treatment of advanced NSCLC, and mutations of the epidermal growth factor receptor (*EGFR*) gene were identified as a crucial factor that correlated with clinical responsiveness to tyrosine-kinase inhibitors (TKI) [5,6]. Gefitinib and erlotinib, which target the EGFR pathway, have been accepted as a superior initial treatment to chemotherapy for patients with *EGFR* mutations and advanced disease [7,8]. Icotinib, which is a potent and selective EGFR-TKI, provides a similar efficacy to gefitinib but with better tolerability for patients with NSCLC previously treated with one or two chemotherapy agents [9,10].

Although four randomized phase III trials that evaluated the efficacy of concurrent erlotinib or gefitinib administration with standard platinum-doublet chemotherapy failed to show evidence of improved survival [11-14], the phase II First-Line Asian Sequential Tarceva and Chemotherapy Trial (FAST-ACT) proved that the sequential administration of TKI following chemotherapy led to a significant improvement in progression-free survival (PFS; *P*= 0.0002) and patients showed a trend toward a better tumor response rate (35.5% versus 24.4%;*P* = 0.12) [15].

Based on the above findings, we designed and conducted this pilot study to assess the efficacy and tolerability of sequential gemcitabine/cisplatin and icotinib administration as an induction therapy for patients with stage IIB to IIIA NSCLC adenocarcinoma.

## Methods

### Study design and eligibility criteria

This was a single-arm study conducted in one center. The primary objective was to assess the objective response rate (ORR), while secondary objectives included safety and perioperative complications, as well as molecular markers for the prediction of tumor response. Patients with histologically confirmed NSCLC adenocarcinoma at stage IIB or IIIA were eligible for enrollment in this study. Other criteria included:age between >18 and <75 years old, an Eastern Cooperative Oncology Group (ECOG) performance status of 0 to 1, and adequate hematological, hepatic and renal function (hemoglobin >10 g/dL, neutrophil count >2.0 × 10^9^/L, platelet count >100 × 10^9^/L, aspartate transaminase (AST; also known as serum glutamic oxaloacetic transaminase) <2.5 × the upper limit of normaland serum creatinine <1.25 × the upper limit of normal). Magnetic resonance imaging (MRI) studies of the brain, ultrasound examinations of the abdomen and supraclavicular lymph nodes, and bone scans were performed to exclude distant metastases. Positron emission tomography-computed tomography (PET-CT) was used as an option for staging. Cardiac and pulmonary function tests were also required before surgery was performed. Patients were excluded if they had received prior chemotherapy, radiotherapy or targeted therapy for any type of malignancy, and patients with interstitial lung disease were also excluded. Informed consent was obtained from patients who participated in the study and the study protocol was approved by the institutional ethics board.

### Study treatment protocol

The chemotherapy regimen consisted of gemcitabine 1,250 mg/m^2^ on days 1 and 8, followed by cisplatin 75 mg/m^2^ on day 1. Subsequently, patients received continuous oral dosing with icotinib (125 mg, three times a day) on days 15 to 28 to complete the 4-week cycle. Toxicity was graded by the National Cancer Institute Common Toxicity Criteria, version 3.0. At the end of two cycles, a repeat computed tomography(CT) was performed to evaluate the response to the induction treatment according to the Response Evaluation Criteria in Solid Tumors (RECIST) criteria. Subsequently, the resectability of each case was carefully assessed by experienced thoracic surgeons, and eligible patients proceeded directly to surgical resection within 2 weeks of the CT assessment. Perioperative complications were also recorded.

### Tumor tissue and plasma samples

Tumor samples from pre-treatment biopsies and surgical specimens were fixed in buffered formalin and embedded in paraffin (FFPE) blocks. Sections from FFPE specimens were stained with hematoxylin and eosin, and the presence of tumor cells was confirmed by a pathologist. Blood samples were collected from each patient before and after the induction treatment, and DNA was extracted from plasma for further analysis.

### Detection of EGFR and KRAS mutations

Genomic DNA was extracted from the FFPE samples using the QIAamp DNA FFPE Tissue Kit (Qiagen, Hilden, Germany). The DNA concentrations were measured by a NanoDrop spectrophotometer (Thermo Scientific, Waltham, MA, USA). The 30 hotspot mutations within exons 18 to 21 of the *EGFR* gene were examined using the Human EGFR Mutation Qualitative Detection Kit (Beijing ACCB Biotech Ltd, Beijing, China). Hydrolysable fluorescent probes were specifically designed to detect different mutations (point mutations, insertions and deletions). The seven hotspot mutations in *KRAS* within codons 12 and 13 were detected by the Human KRAS Mutation Qualitative Detection Kit (Beijing ACCB Biotech Ltd, Beijing, China). This experiment was performed using the MX3000P PCR system (Stratagene, La Jolla, CA, USA) according to the manufacturer’s protocol. The PCR conditions were as follows: initial denaturation at 95°C for 10 minutes, followed by 40 cycles at 95°C for 15 seconds and 60°C for 1 minute.

### Denaturing high-performance liquid chromatography (DHPLC) analysis

As described previously [16], *EGFR* (exon 19 and 21) mutations were detected in plasma using DHPLC Transgenomic Wave Nucleic Acid Fragment Analysis System (Transgenomic, Omaha, NE, USA).

### Quantitative real-time PCR

Total RNA samples from the FFPE tissue sections were isolated by an RNeasy FFPE Kit (Qiagen) according to the manufacturer’s instructions. The concentrations of total RNAs were evaluated on the microspectrophotometer (OD260/230 ≥1.7; OD260/280 = 2.0 ± 0.2). The RNA samples were diluted to the working concentration of 20 ng/μLto 200 ng/μL. The expression levels of ERCC1 and RRM1 were examined using the Human ERCC1 and RRM1 Expression Relative Quantitative Detection Kits (Beijing ACCB Biotech Ltd). The reverse transcription was performed at 25°C for 10 minutes, followed by 37°C for 60 minutes. TaqMan probes were used to detect the expression levels of *ERCC1* and *RRM1*. *ACTB* (encoding β-actin) was used as the internal control. The PCR amplification was performed on the MX3000P (Stratagene) and the procedures were as follows: initial denaturation at 95°C for 10 minutes, followed by 40 cycles at 95°C for 15 seconds and 60°C for 1 minute. The relative amount of target genes was normalized to the internal reference gene (*ACTB*) using the 2^-ΔCt^ method (ΔCt = Ct_target gene_-Ct_ACTB_). The relative expression level of *ERCC1* and *RRM1* was then categorized as high, moderate or low.

### Immunohistochemistry

Briefly, 4-μm paraffin sections from paraffin blocks of clinical samples were prepared. After deparaffinization, the sections were treated with hydrogen peroxide (H_2_O_2_) for 5 minutes to block the endogenous peroxidase. These sections were stained with the mouse monoclonal anti-ERCC1 antibody (ZM-0138; ZSGB Biotech Ltd, Beijing, China) and anti-RRM1 antibody (60073-1-1 g; Proteintech Group, Chicago, IL, USA). The tumor was considered to show positive expression when more than 10% of tumor cells were stained at any intensity.

### Statistical analysis

The primary end point was the ORR according to the RECIST criteria. On the basis of a 45% response rate with a 95% confidence interval (CI) equal to the response rate plus or minus 30%, a sample size of 11 patients was estimated as necessary. The objective of statistical analysis was to detect the degree of correlation between the radiographic response to the induction treatment and the presence of an *EGFR* mutation. Statistical analysis was performed using patched SPSS 17.0 for Windows software (SPSS Inc, Chicago, IL, USA). Data were tested using Fisher’s exact test and the analysis results were considered significant at a level of *P*< 0.05.

## Results

### Patient characteristics

A total of 11 patients were enrolled in this study between August 2011 and April 2012 at a single department of Peking University Cancer Hospital (Beijing, China). The clinical characteristics of the patients are listed in Table 1. The patients included five males and six females, and most patients were identified as having stage IIIA disease before treatment; three patients had a bulky N2 metastasis. The majority of patients were diagnosed with adenocarcinoma (10 out of 11 patients), while one patient was diagnosed with adenosquamous carcinoma postoperatively.

**Table 1:** Patient characteristics before treatment

**Variable**	**Median (range) or frequency (%)**
Age (years)	55 (42 to 61)
Gender	
Male	5(45%)
Female	6(55%)
Smoking status	
Current	4(36%)
Former	1(9%)
Never	6(55%)
Location of the primary tumor	
Central	2(18%)
Peripheral	9(82%)
Biopsy method	
Bronchoscopy	3(27%)
CT-guided needle biopsy	6(55%)
EBUS-TBNA	2(18%)
NSCLC clinical stage	
IIB	2(18%)
IIIA	9(82%)

CT, computed tomography; EBUS-TBNA, endobronchial ultrasound-guided transbronchial needle aspiration; NSCLC, non-small-cell lung cancer.

### Treatment delivered and toxicity

All 11 patients enrolled in the study completed two cycles of sequential treatment and six patients went on to undergo surgical resection. The median time from the start of treatment to surgery was 71 days, and the median time from the last dose to surgery was 14 days. The toxicities that occurred during the induction therapy are listed in Table 2. Most of the adverse events were grade 1 or 2, while only one case of grade 3 neutropenia was recorded. Common toxicities included neutropenia, alanine transaminase (ALT) elevation, fatigue, dry skin, rash, nausea, alopecia and anorexia. Neither dose reduction nor discontinuation was required in the induction cycles.

**Table 2:** Toxicities related to the study treatment

**Toxicity**	**Grade**				**Total**
	**1**	**2**	**3**	**4**	
Hematological					
Leukopenia	3	2	0	0	5/11 (45%)
Neutropenia	2	2	1	0	5/11 (45%)
Anemia	2	0	0	0	2/11 (18%)
Thrombocytopenia	2	1	0	0	3/11 (27%)
Biochemical					
Increased ALT	6	0	0	0	6/11 (55%)
Non-hematological					
Rash	4	3	0	0	7/11 (64%)
Dry skin	4	2	0	0	6/11 (55%)
Pruritus	3	0	0	0	3/11 (27%)
Alopecia	6	2	0	0	8/11 (73%)
Mucositis	2	0	0	0	2/11 (18%)
Fatigue	5	2	0	0	7/11 (64%)
Anorexia	7	0	0	0	7/11 (64%)
Pyrexia	0	0	0	0	0/11 (0%)
Weight loss	0	0	0	0	0/11 (0%)
Nausea	9	0	0	0	9/11 (82%)
Vomiting	2	1	0	0	3/11 (27%)
Diarrhea	2	0	0	0	2/11 (18%)
Constipation	5	0	0	0	5/11 (45%)

ALT, alanine transaminase.

### Treatment efficacy and surgical outcomes

After two cycles of sequential administration, 5 out of 11 patients achieved a partial response (PR) according to the RECIST criteria resulting in a response rate of 45%, but no complete response (CR) was observed. Among the six other patients, five patients had stable disease (SD) and one patient had progressive disease (PD) owing to the appearance of a supraclavicular lymph node metastasis. After the induction therapy was evaluated, all five patients with PR and the one patient with SD underwent surgery; while another three patients with SD and bulky mediastinal lymph node metastases received pemetrexed for chemotherapy owing to the unresectability of their disease. One patient with SD refused to undergo resection and opted for radiotherapy. The patients with PD also received radiotherapy.

For the six patients who received surgery, five patients underwent a complete lobectomy combined with systematic mediastinal lymph node dissection, and the other patient underwent a thoracotomy and exploration; pleural dissemination (M1a) was found and the operation was halted. Among the five lobectomies, two patients had a bronchoplasty and no angioplasty was performed. The postoperative pathological examinations confirmed that the diagnosis was consistent with the pre-treatment biopsy in all cases except one adenosquamous carcinoma, and no pathologic complete response (pCR) was noted. Two cases were down-staged by means of induction therapy, and another two patients who had been previously diagnosed stage IIB were later diagnosed to be mediastinal lymph node metastasis (stage IIIA). Postoperative complications included one case each of atelectasis, supraventricular arrhythmia and wound infection. No intraoperative hemorrhage or postoperative death was observed.

### Molecular marker analyses

*EGFR* mutation analysis was performed on biopsy samples of nine patients before induction therapy, two of whom had exon 19 deletions. After surgery, specimens from six patients were available for analysis and three patients were confirmed to harbor exon 19 deletions. Neither exon 18, 20, 21, nor *KRAS* mutations, were detected in this cohort of patients. An increased trend toward achieving a clinical response was noted in patients with *EGFR* mutations, although the difference was not statistically significant (*P*= 0.083). Plasma samples of 11 patients were collected before treatment in addition to the six surgical samples after induction therapy. However, no *EGFR* mutations were detected in the plasma samples collected according to the DHPLC method.

Expression analysis was only performed in resected tumors, and we observed that five out of six patients had high mRNA levels of *RRM1* and low levels of *ERCC1*. All of these patients were scored as *ERCC1*-negative and three patients were *RRM1*-positive according to immunohistochemistry, respectively. All of the molecular data and treatment outcomes are listed in Table 3.

**Table 3:** Treatment outcomes for patients associated with molecular marker status

**Patient**	**Gender**	**Smoking status**	**EGFR status**				**ERCC1**		**RRM1**		**CR**
			**Biopsy**	**SS**	**Pre-Pl**	**Post-Pl**	**mRNA**	**IHC**	**mRNA**	**IHC**	
1	M	Current	Wild	Ex19 Del	Wild	Wild	+	(−)	+++	(+)	PR
2	F	Never	Wild	/	Wild	/	/	/	/	/	SD
3	F	Never	Wild	/	Wild	/	/	/	/	/	SD
4	M	Current	/	Wild	Wild	Wild	+	(−)	+++	(−)	SD
5	F	Never	Wild	Wild	Wild	Wild	+	(−)	+++	(−)	PR
6	F	Never	Wild	Wild	Wild	Wild	+	(−)	+++	(+)	PR
7	F	Never	Wild	/	Wild	/	/	/	/	/	SD
8	F	Never	Ex19 Del	Ex19 Del	Wild	Wild	+	(−)	+++	(+)	PR
9	M	Current	Wild	/	Wild	/	/	/	/	/	PD
10	M	Current	Ex19 Del	Ex19 Del	Wild	Wild	++	(−)	++	(−)	PR
11	M	Former	/	/	Wild	/	/	/	/	/	SD

CR, clinical response; EGFR, epidermal growth factor receptor; F, female; IHC, immunohistochemistry; M, male; PD, progressive disease; post-Pl: post-treatment plasma; PR, partial response; pre-Pl: pre-treatment plasma; SD, stable disease; SS, surgical specimen.

## Discussion

To our knowledge, this is the first study to use sequential treatment as an induction therapy for NSCLC, and ORR was our primary outcome measure. The CHEST study achieved a response rate of 35.4% with gemcitabine and cisplatin as the induction regimen. Three clinical trials have evaluated the efficacy of preoperative EGFR-TKI, all of which enrolled patients with stage I to II NSCLC. The ORRs of these studies were unstable, ranging from 0% to 42% [17-19]. In our study, we observed a response rate of 45% in patients with stage IIB to IIIA NSCLC, which was comparable to previous studies of induction therapy use for patients with early stage disease and for advanced disease in the FAST-ACT trial (ORR = 35.5%). In addition, the sequential administration of icotinib and chemotherapy was well tolerated with no serious adverse eventsrecorded perioperatively. This was a better result than that of previous studies, especially with regards to rash and diarrhea. This result was consistent with the ICOGEN trial, which showed that icotinib caused less toxicity than gefitinib, although the number of cases in this study is too limited to make definite conclusions in this regard [10].

It is unclear whether the targeted therapy or chemotherapy plays the most important role in achieving clinical response in each patient. However, an *EGFR* mutation is one of the most important factors to be considered. In the FAST-ACT study, six samples were available for the *EGFR* mutation analysis from patients who received sequential treatment, of which two patients had exon 19 deletions and achieved a PR. Among 11 patients, we observed that all three cases with an exon 19 deletion had a clinical response after induction therapy; for the seven patients with wild-type *EGFR*, only two met the criteria for a response. This result was consistent with previous studies regarding gefitinib induction therapy [19]. Although only a non-significant trend was observed in favor of patients harboring *EGFR* mutations (*P*= 0.083), we believe that the difference would be significant if more patients were enrolled and unselected for other molecular markers.

It has been proven that *RRM1* and *ERCC1* mRNA levels and protein expression are predictive of response to gemcitabine and platinum-based chemotherapy [20,21]. In our study, most of the patients who had achieved a clinical response had low levels of *ERCC1* mRNA and high levels of *RRM1* mRNA, which was similar to the postoperative immunohistochemistry results. One possible explanation is that RRM1 expression status might not be the crucial factor in this sequential treatment. In addition, although Bepler *et al*. concluded that post-chemotherapy gene expression levels were representative of pre-treatment levels, the analysis of 10 cases may not be sufficient. Furthermore, it has been noted that gene expression levels increase after chemotherapy, which indicates that gemcitabine may alter *RRM1* mRNA levels [22]. To confirm this theory, a further study is needed to assess patients, with a radiographic response in particular.

Since *EGFR* mutation status has been considered to be a predictor of response to EGFR-TKI, the identification of patients with an *EGFR* mutation is important for the use of sequential induction therapy. Tumor tissue is the best choice for *EGFR* mutation analysis and these enable the microdissection of tumor cells. In our study, the postoperative samples of three patients were found to have exon 19 deletions, one of which could not be detected in the analysis of biopsy tissue preoperatively. Intratumor heterogeneity for *EGFR* mutation has been proven to exist by the analysis of different areas of a single NSCLC tumor [23], and further evidence of this heterogeneity stems from the predictive benefit of EGFR-TKI treatment from the relative abundance of *EGFR* mutations [24]. However, the difference in treatment efficacy between patients with and without heterogeneity appears to lie with PFS and OS, rather than the radiographic response, and the patient in our study also achieved a PR. When insufficient biopsy tissue or tumor cells are available for mutation analysis, the detection of mutations in plasma DNA might be an alternative. It has been reported that approximately 80% of mutations could be detected in both the plasma DNA and the corresponding tumor samples in patients with stage IIIB to IV disease [16]. However, in our analysis, no *EGFR* mutation was detected in any plasma samples, either pre- or post-treatment. This negative result may have been due to differences in the amount of circulating DNA derived from tumor tissues of patients with stage IIB to IIIA and advanced-stage NSCLC.

## Conclusion

Two cycles of sequentially administered gemcitabine/cisplatin with icotinib as the induction treatment is a feasible and efficacious approach for stage IIB to IIIA NSCLC adenocarcinoma. This regimen showed a promising ORR with no observed additional toxicity or postoperative complications, but this should be confirmed by studies with larger sample sizes. *EGFR* mutations appeared to be a predictor for response, which will be confirmed by further studies that may identify other molecular markers of clinical response as a result of sequential therapy.

## Consent

Written informed consent was obtained from the patients for publication of this report and any accompanying images.

## Abbreviations

ALT: alanine transaminase; AST: aspartate transaminase; ChEST: Chemotherapy in Early Stages Trial, CI, confidence interval; CR: complete response; CT: computed tomography; DFS: disease-free survival; DHPLC: denaturing high-performance liquid chromatography; EBUS-TBNA: endobronchial ultrasound-guided transbronchial needle aspiration; ECOG: Eastern Cooperative Oncology Group; EGFR: epidermal growth factor receptor; ERCC1: excision repair cross-complementing rodent repair deficiency complementation group 1; FAST-ACT: First-Line Asian Sequential Tarceva and Chemotherapy Trial; FFPE: formalin and embedded in paraffin; HR: hazard ratio; IHC: immunohistochemistry; KRAS: v-Ki-ras2 Kirsten rat sarcoma viral oncogene homolog; LACE: Lung Adjuvant Cisplatin Evaluation; MRI: magnetic resonance imaging; NSCLC: non-small-cell lung cancer; ORR: objective response rate; OS: overall survival; PCR: polymerase chain reaction; pCR: pathologic complete response; PD: progressive disease; PET-CT: positron emission tomography-computed tomography; PFS: progression-free survival; PR: partial response; RECIST: Response Evaluation Criteria In Solid Tumors; RRM1: ribonucleoside-diphosphate reductase large subunit; SD: stable disease; TKI: tyrosine-kinase inhibitor.

## Competing interests

The authors declare that they have no competing interests.

## Authors’ contributions

CL participated in the design of the study, performed the statistical analysis and drafted the manuscript. YYM, QF, FF, HB and BZ participated in study design, literature search and coordination. SL, JF, SY, NW, QZ, JC, JW, YF, YW and YP participated in the analysis of experimental results. YY conceived of the study, participated in its design and coordination, and helped to draft the manuscript. All authors read and approved the final manuscript.
